# Intussusception, Etc.

**Published:** 1872-05

**Authors:** Alfred W. Taliaferro

**Affiliations:** San Rafael, Cal.


### Selections.

*Intussusception—Novel Treatment—Success.* By ALFRED W. TALIAFERRO, M.D., of San Rafael, Cal.

The following case of invagination of the gut, occurring in the State Prison Hospital, is sent because of the novelty of the treatment adopted and the success attending it. On June 8, 1871, in making my usual visit to the prison, I found in hospital, T. S., æt. 24, admitted fourteen hours previously, apparently suffering from a severe cramp colic, and from which he had obtained no relief. Not doubting but that this was a simple case, I ordered the usual remedies without making a very careful examination. The next day he was still suffering intensely and vomiting; there had been no action of the bowels, although the most active medicines had been given by the mouth and as enemata ; the hypodermic administration of a grain of morphine had given him a few hours of sleep and ease.

A careful examination revealed a mechanical obstruction in the bowels which I diagnosed as being at the ilio-cæcal valve, the intense pain being confined to that spot, and an examination per anum demonstrated that the rectum and sigmoid flexure of the colon were free. All efforts to remove this obstruction proved abortive. During the evening he commenced vomiting large quantities of fæcal matter.

On the 11th, he was in a state of collapse, and I returned to San Rafael with no hopes of finding him alive at my visit the next morning; therefore I was greatly surprised on my return to find him not only alive, but in a comfortable condition, and with free evacuations from the bowels! This favorable change was due to the treatment adopted by Wm. Lebur, my steward in the hospital. Lebur reported that as everything directed by me had failed to relieve the patient, and in consequence of his desperate condition, he had ventured, on the evening before, the experiment of inflating the intestines with the *effervescing draught*, in order that the obstruction might be overcome! This was effected by injecting into the rectum a strong solution of bicarbonate of soda, then a strong solution of tartaric acid, and preventing its egress by making rigorous pressure against the sphincter ani. The result of this experiment was the instant removal of the invagination, and free catharsis.

The principle of this treatment becomes obvious when we reflect a moment, and I wonder that it has not been in general use. Our chief aim, in this disorder, being to remove obstruction by inflation of the bowels, what method is there more potent than the one used in this instance?*

Lebur is a U. S. prisoner from Oregon, and although he had practiced medicine in that State before he became a prisoner, he is by no means a "regular." He claims this treatment as original; and as far as he is concerned, I believe that it did originate with him on this occasion.

* Historical note by Editor. In 1726, Dean Swift wrote Gulliver's Voyage to Laputa. The description of Gulliver's visit to the Grand Academy at Lagado is graphic; among other things, he says: "I was complaining of a small fit of the colic, upon which my conductor led me into a room where a great physician resided, who was famous for curing that disease by contrary operations from the same instrument. He had a large pair of bellows, with a long slender nozzle of ivory; this he conveyed eight inches up the anus, and drawing in the wind, he affirmed he could make the guts as lank as a dried bladder. But when the disease was more stubborn and violent, he let in the nozzle while the bellows were full of wind, which he discharged into the body of the patient, then withdrew the instrument to replenish it, clapping his thumb strongly against the orifice of the fundament; and this being repeated three or four times, the adventitious wind would rush out, bringing the noxious along with it (like water put into a pump), and the patient recovered. I saw him try both experiments upon a dog, but could not discover any effect from the former. After the latter, the animal was ready to burst, and made so violent a discharge as was very offensive to me and my companion. The dog died on the spot, and we left the doctor endeavoring to recover him by the same operation."

We fear, from the last sentence, that the "great physician" was homœopathically inclined.—Ed.

On consulting various authorities, I find only in "Wood's Practice of Medicine," third edition, any account of similar treatment. It is a simple mention of a report made by Dr. Tate, in the *Georgia Southern Medical Journal*, of a successful case treated in this manner.

Lebur's reading is limited, and I must believe his assertion that his action in this case was not based on any information he had received from books or hearsay. I give *literatim* a portion of the report of this case he made me in writing, because for style, for reasoning from cause to effect, and for other little disquisitions, there is nothing in our medical literature so unique:

"At 9 oclock at night June 11th I gave the following prescription R

| | | | |
|---|---|---|---|
| | Carbonate of Soda . . | oz iv | |
| | Aqua . . . . . | Oij. | |
| Also R | Tartaric Acid . . . | dr ij | |
| | Aqua . . . . | oz xviij | Mix. |

Sigma; First inject one third of the lixivium in the rectum. then before withdrawing the Syringe grasp a sponge in the hand and press it with considerable force against the fundiment to prevent the return of the effervescing gass untill the Patient complains of pain or extreme anxiety to evacuate the bowels or rather untill the abdomen becomes considerably distendid. it will then be necessary to remove the sponge immediately. at which time there will be a profuse evacuation of the bowels: if Eructations from the Stomach or Singultus, or Cramping of the bowels Supervene the abdomen Should be immediately bandaged with very moderate force, otherwise the Counter pressure mite cause an eruption of the bowels or wholly impeed respiration by the upward pressure against the Diaphragm. in the case under consideration the patient ejected wind from the Stomach and began to hiccough and water ran from the eyes. now the question arrises whether the effervescence passed through the ilio-colic valve or not, or whether the distendid and upward pressure of the bowels against the Stomach and Diaphragm. caused the belching and hiccough or not; the Patient could not breathe untill the Sponge was removed to give vent to the Enema which was repeated three-times within half an hour. one third of the foregeing amount Specified was given at each injection as above mentioned. Since the time the enema was given on the night of the 11th the patient has had evacuations of the bowels two or three-times a Day until recently: they are now regular once a Day. he appears very weak Pulse averageing about 100; appetite good.

(Signed) "Wm. Lebur."

"To Dr. Taliaferro."

I, of course, would not suggest such heroic doses as were given here, for there is no wonder that the tears ran from this poor fellow's eyes; but would advise a trial of this plan in a more conservative way. I have had occasion since to try it with a female prisoner in a case of obstruction, supposed to be either from invagination or a twist in the intestine, but the case was not sufficiently pronounced for me to feel sure that it proceeded from either of these causes. For two days I tried the most active purgatives and enemata without any effect; remedies were also used for allaying spasm of the bowels; she suffered intense pain over the whole abdomen, but there was no vomiting. With the light of Lebur's experiment before me, I ordered the trial of the effervescing mixture, but not in such *blasting* doses. A pint of water with two drachms of soda, and the same amount of water with a drachm of the acid, were alternately introduced into the rectum. In an hour there was a copious evacuation from the bowels, and the woman had no further trouble.

I hope the publication of these cases may induce others to try this mode of treatment, as I feel sure it will prove more efficacious than any other that has been tried in this very fatal disorder.

The poor fellow, T. S., had slight diarrhœa for a few days, was relieved and seemed in a fair way to recover, but inflammation of the parotid gland set in, extensive suppuration ensued, and he sank from exhaustion on the 25th June. Could this condition of the glands have been accidental? It is more rational to assume that there was inflammation of the gut near its seat of trouble; that exudation of morbific matter followed as a result, and, entering the circulation, had acted as a blood poison on these glands. There was no other evidence of pyæmia in this case; none of the other glands of the body were affected, and there was no redness or inflammation along the course of any of the veins.

I regret that I was absent from the county at the time of his death, as a *post mortem* would probably have fully settled the question.—*San Francisco Western Lancet.*

